# Modeling the effect of autonomous vehicles (AVs) on the accessibility of the transportation network

**DOI:** 10.1038/s41598-024-60069-8

**Published:** 2024-04-23

**Authors:** Hamid Mirzahossein, Mahdis Mashhadloo

**Affiliations:** Department of Civil – Transportation Planning, Faculty of Technical and Engineering, Imam Khomeini International University (IKIU), Qazvin, Iran

**Keywords:** Autonomous vehicles, Transportation network, Accessibility, Hybrid heuristic assignment algorithm, Civil engineering, Engineering

## Abstract

The utilization of autonomous vehicles (AVs) has emerged as a pivotal factor in addressing the rising costs and safety concerns associated with modern travel. As technology advances and traffic challenges intensify, enhancing accessibility stands out as a critical goal for transportation experts. Accessibility, constrained by factors like travel time, underscores the increasing need for AVs to mitigate these limitations. This study aimed to model the influence of AVs on the accessibility index within transportation networks and discuss system optimization based on user equilibrium (UE) and system optimum (SO) outcomes. The research conducted numerical analysis employing the Hearn network as a fundamental system to validate a mixed assignment model and ascertain baseline accessibility. Additionally, the Sioux Falls network, a medium-sized network, was employed for analysis. A hybrid heuristic assignment algorithm was introduced, concurrently assigning different percentages of AV presence alongside the remaining non-AV percentage in three distinct scenarios. These scenarios ranged from 0 to 100% AV presence: the first scenario maintained constant network capacity, the second scenario adjusted network capacity based on AV presence, and the third scenario incorporated capacity adjustments in the assignment stage. In all three scenarios, network accessibility was evaluated using gravity and accessibility index methods derived from the hybrid assignment model output. The findings demonstrated that as the percentage of AVs increased, accessibility improved in both Hearn and Sioux Falls networks across all scenarios. The second and third scenarios exhibited higher accessibility increases compared to the first, attributable to augmented capacity resulting from increased AV presence. In the Sioux Falls network, the first scenario showed enhanced SO and UE due to increased AV presence and enhanced system operator management. Conversely, the second and third scenarios, with increased AVs and subsequent capacity increments, displayed reduced UE and SO results. Despite the decline in UE and SO, traffic flow assignment and overall network accessibility improved. These findings highlight the positive correlation between AV presence, network capacity, and enhanced accessibility. The study underscores the potential benefits of AV integration in optimizing transportation networks and improving overall accessibility, albeit with nuances in capacity adjustments impacting traffic flow dynamics. Further research avenues could explore complex traffic flow scenarios and delve into more specific optimization strategies.

## Introduction

Autonomous vehicles (AVs) are vehicles that can cover all aspects of driving in any environmental condition. According to Litman's research, the use of AVs started before 2020, and the expansion of their use will probably be realized in 2040^1^. AVs promise a fundamental change in transportation. Travel is expected to be safer, cheaper, easier, and more sustainable through the use of AVs, and as a result, travel costs will also decrease^2,3^. According to estimations, AVs will have a special place in urban and intercity transportation in the next ten years. It is clear that with the application of these vehicles and their positioning, the planning horizons for the coming years will change. Modeling tools give researchers the possibility of impact measurement in transportation networks and make it possible to understand the future better. Among the effects of AVs, increasing safety, reducing fuel consumption, and creating a happier and more active life for people can be mentioned. The development of AVs can also be imagined under the influence of the desire of large companies to invest and take over the market share.

AVs can identify their surroundings and move safely alone or with little assistance^4,5^. Different autonomy levels are envisioned for such vehicles^6,7^. AVs make it possible for those who are unable or unwilling to drive to move by themselves without needing a driver. These people include the disabled and elderly, or young people without a certificate. As a result of this capability, more destinations are available to these groups and the number of trips they can make will increase^8^. On the one hand, cheap trips and the competition created between shared and private vehicles provide the opportunity to access more distant destinations, including the suburbans, for this group of users^9^. Studies in the field of people’s desire to use AVs in different categories indicate that the degree of desire depends on factors such as gender, income, age, etc.^10^. On the other hand, shared AVs provide the possibility of multiple trips at a low cost and without worrying about the costs of owning a vehicle (such as depreciation, insurance and taxes, and repairs)^11^. In addition, it is possible for the passengers of such vehicles to do other activities unrelated to driving. Such an approach will certainly cause more users to favor such vehicles, and as a result, it will lead a wave of people to use such vehicles^12^.

Each vehicle can benefit from information obtained from other vehicles in its vicinity, especially information about traffic congestion and safety hazards. Vehicular communication systems use vehicles and roadside units as communication nodes in a peer-to-peer network and provide information to each other^13^. In a cooperative approach, vehicular communication systems can cooperate with all vehicles to be more effective. According to a study conducted by the National Highway Traffic Safety Administration, vehicular communication systems can prevent up to 79% of traffic accidents^14^.

Significant research has supported both the potential benefits of AVs in enhancing travel efficiency and the possibility of increased travel demand resulting from their adoption. AVs are equipped with communication systems that allow them to communicate with other AVs and roadside units to provide them with information about the road or traffic congestion. In addition, scientists believe that the future will be accompanied by computer programs that will manage each private vehicle as it passes through the intersection. This type of connection can replace traffic lights and stop signs. These types of features also create and develop the ability of AVs to cooperate with other services (such as intersection computer systems) in the AV market. This issue can lead to creation of a network of AVs that all use the same network and the information in that network. Finally, the application of this problem can lead to more use of AVs in the network because the information is verified by the use of other AVs. Such movements strengthen the value of the network, and these movements are called external factors of the network^15^.

In transportation planning, accessibility refers to a measure of ease of approaching and interacting with a destination^16^ or activities performed in a space, for example, around a city or country^17,18^. The transportation system must provide access to all administrative, medical, and service destinations, and this is not only done by approaching the destinations in the network but also requires increasing the efficiency of the transportation system^19^. Hansen first proposed the term accessibility as a concept in the field of transport quality in a region^20^. Also, according to the definition of Bertolini et al., accessibility is usually defined as the number of places that can be accessed in a certain travel time or cost^21^.

The concept of accessibility plays an important role in several scientific fields, for example, transportation planning, environmental protection, economic development, etc., and changes in accessibility can have a direct impact on a person’s quality of life. Transportation accessibility is a function of connectivity between an origin (e.g. a home) and a destination (e.g. a workplace). In order to evaluate accessibility, criteria have been proposed by Geurs et al., which include theoretical foundations, interpretability, operationality, and usability^22^. On the other hand, accessibility is associated with land use. Increasing accessibility in a certain location increases land development's desirability^23^. This relationship is often used in transportation and land use forecasting models. Geurs et al. identified four components from different definitions of accessibility in theory and practice. These components include land use, transportation, time, and person^22^. Therefore, accessibility should be related to travel opportunities and land use changes. This should also be considered in connection with personal accessibility. In addition, the accessibility paradigm considers both mobility and proximity factors as part of itself, in close connection with each other^24^. In fact, adding more density to areas can lead to less congestion, but developments in AVs industry can reduce accessibility over a long period of time by creating more AV trips that increase dependence on vehicles^25^.

Fully AVs are expected to change accessibility fundamentally. From the point of view of transportation, such vehicles are expected to minimize human errors and make travel much more reliable^26^. Moreover, such vehicles completely reduce travel costs^27^, make it safer^28^, provide more comfort^29^, and make trips better from an environmental point of view. These vehicles also make vehicular travel possible for children, the elderly, and the disabled^30^. Based on the different scenarios expressed in studies, the presence of AVs can reduce the total number of transport fleets^2,31,32^. If these assumptions are implemented, AVs will not only revolutionize transportation but also change the shape of the city, and by reducing the overall costs of travel, they will also increase the total demand for travel and cause a new wave of urban marginalization and urban expansion^33^. AVs are expected to offer greater convenience at lower prices while increasing road capacity. This is the same pattern that reminds the increase of private vehicles and, later, the construction of highways. Based on the operational definitions, the demand-to-cost factor is introduced as an accessibility index. Therefore, in this research, related and influencing factors on the demand and cost, as well as accessibility, were investigated in order to measure the effectiveness of accessibility due to the use of AVs.

Meyer et al. (2017) simulated the effect of AVs on accessibility by the use of national transport models of Swiss municipalities. It was revealed that AVs could lead to another quantum leap in accessibility. Also, spatial distributions of accessibility effects indicated that AVs favor urban sprawl and can make public transportation redundant except for dense urban regions^34^. Luo et al. evaluated the effect of AVs on accessibility by the use of agent-based disaggregate simulations (MATSim). This study was conducted on Japanese regional cities using a spatial dataset of persons’ trip surveys. Shared and private AVs and human-driven private cars were applied in the simulation as two autonomous transport modes. A remarkable market share of AVs was indicated in the scenarios, and when private AVs were presented in the scenario, the overall accessibility was increased. Further accessibility gains were represented in suburban areas that may lead to more urban sprawl in the future^35^. Zhong et al. evaluated the long-term impacts of congestion pricing, as well as shared AVs, on job accessibility, transportation, and land, use. They revealed that introducing shared AVs reduced the negative effects of road congestion pricings and contributed to population development as well as job in charging zones^36^. Dianin et al. conducted a study to represent a framework for important and developing aspects regarding AV implication of accessibility as well as transport equities. They discussed four accessibility effects of AV based on the literature, including social accessibility inequity mitigation, social accessibility inequity exacerbation, accessibility sprawls, and accessibility polarizations^37^. Eppenberger and Richter examined the opportunity of shared AVs for improving spatial equities of accessibility as well as socioeconomic developments of four European urban cities using a linear regression model. Results indicated the positive effect of educational attainments on accessibility in all areas. Also, for London as well as Paris, the positive effect of yearly incomes on accessibility was represented, and conversely, in Vienna and Paris, the increased unemployment rate had a negative effect. Moreover, the potential of shared AVs was evident in all areas by comparing car and public transportation accessibilities^38^. Petrović et al. investigated differences in AV acceptances in physically disabled people using the Bayesian linear regression model. It was indicated that the main aspects of AV successful introductions were trust, accessibility, and attitudes. Also, AVs represented the great potential for improving accessibility to transportation and ensuring transportation equities^39^.

Due to the fact that today the discussion of using AVs has been widely favored by manufacturers and consumers, it is expected that the development of such vehicles will become more popular in the future. Therefore, investigating the characteristics and the effect of such devices on the intra-city transportation network can greatly affect future urban planning, especially in the field of accessibility as one of the important indicators of transportation. Therefore, the impact of AVs on accessibility is analyzed and investigated in this research.

Many believe that the presence of AVs will have a great impact on the urban lifestyle. The increase in the presence of people in cities causes an increase in the share of traffic in cities and, as a result, a decrease in accessibility. From this point of view, it becomes necessary to facilitate people's accessibility in cities. Therefore, it is required to study the impact of such vehicles on the future of transportation and especially accessibility. On the other hand, accessibility is one of the most important transportation issues nowadays. Due to the many restrictions on accessibility, such as travel time, etc., the need to use AVs, which reduce the restrictions, is felt more. To date, a limited number of studies have been conducted around the impact of AVs on accessibility, and considering the above-mentioned, more research is needed in the field of AVs. Therefore, the main goal of this study is to model the impact of AVs on the accessibility index in the transportation network. Also, the simultaneous solution of the assignment problem of AVs and non-AVs using a hybrid heuristic assignment method and improvement in the assignment of transportation network traffic as the innovation of this research was investigated.

## Methodology

**Figure 1:**
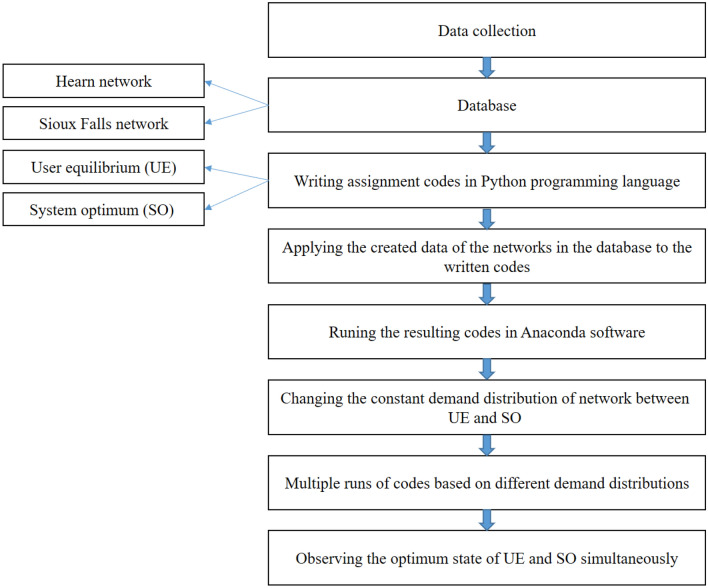
Steps used in this study.

In this research, the required data were selected using Hearn and Sioux Falls networks. Also, a hybrid heuristic assignment algorithm was implemented, which simultaneously assigned and analyzed different percentages of the presence of AVs along with the remaining percentage of non-AVs in the network in the form of three scenarios. Then the network accessibility was studied through two different calculation methods of gravity and accessibility index with the information obtained at the end of each assignment stage. These scenarios include different modes of capacity calculation in the network. In order to analyze this information, Anaconda software was used, in which all steps are coded with the Python programming language. The geographical area used in this research was adapted from the Sioux Falls transportation network (located in South Dakota, USA). Figure 1 shows the steps of the research method.

### Data collection

In this section, the required data was obtained from the Hearn and Sioux Falls networks, and in order to code and facilitate the network assignment, the Python programming language was used.

### Traffic assignment

Traffic assignment refers to the selection of routes between the origin and destination in transportation networks; A process to estimate the flow in the transportation network for a given demand. In fact, its purpose is to assign the origin–destination matrix to the network. Traffic assignment is the fourth step of the conventional transportation forecasting model, which is done after trip generation, trip distribution, and modal split. To determine the needs of facilities and the associated costs and benefits, the number of passengers per route and network link must be known (a route is simply a chain of links between an origin and a destination). As a result, traffic assignment in the network is very important^40^.

Due to the existence of two types of AV and non-AV traffic flows, two traffic assignment methods were used, the system optimum (SO) assignment method for AVs and the user equilibrium (UE) method for non-AVs. It is important to mention that the SO assignment method can be applied in the case that AVs are in the optimum state and in communication with each other. Also, the written assignment codes perform two types of SO and UE assignments simultaneously.

#### UE

What gives rise to the concept of equilibrium in transportation networks is that the travel time of network links is not a constant value but is a function of the flow in that link, and the travel time increases with the increase of the flow in a link. This issue causes an interaction between the number of users of different routes and their travel time, which creates the equilibrium problem and obtaining an equilibrium flow. When the flow passing through the links of a network is known, it can be used to evaluate the network, determine the service level of the links, design issues, control, policy making, tolls, etc.^41^.

The equilibrium flow in the network results from the accumulative or simultaneous decisions of all users. It would be reasonable to assume that each driver tries to minimize the travel time between the origin–destination pair. Of course, this does not mean all passengers of the same origin–destination will use a specific route. A stable flow will occur when no travelers can unilaterally improve their travel time by changing their routes. This statement is commonly referred to as UE condition or Wardrop’s principle. In other words, in equilibrium, for each origin–destination pair, the travel time for all used routes is equal and less than or equal to the travel time of unused routes. Therefore, according to this principle, in UE condition, some of the available routes between each origin–destination may be used and some may not be used, and if there are several used routes, the travel time of all of them will be equal, and all the routes that the passengers of that origin–destination do not use must have a travel time of at least as much as the used routes^42^. The equilibrium flow pattern in links can be obtained by solving Eq. (1):

1 $${\text{min}}z\left(x\right)= {\sum }_{a}{\int }_{0}^{{x}_{a}}{t}_{a}\left(\omega \right)d\omega ,$$

$${\sum }_{k}{f}_{k}^{rs}= {q}_{rs} \forall rs$$

$${x}_{a}= {\sum }_{rs}{\sum }_{k}{f}_{k}^{rs}{\delta }_{a.k}^{rs} \forall a$$

$${f}_{k}^{rs} \ge 0 \forall k. rs$$

where the objective function ($z\left(x\right)$) is the sum of the definite integrals of the performance functions of the network links, ${t}_{a}\left(\omega \right)$ is the travel time of link $a$, ${f}_{k}^{rs}$ is the flow on path $k$ connecting origin $r$ and destination $s$, ${q}_{rs}$ is the demand for travel from $r$ to $s$, ${x}_{a}$ is traffic volume of link $a$, and ${\delta }_{a.k}^{rs}$ is an indicator variable taking a value 1 if link $a$ is included in path $k$ between $r$ and $s$, and 0 otherwise^43,44^.

The restrictions ${\sum }_{k}{f}_{k}^{rs}= {q}_{rs}$ are so-called traffic flow conservation restrictions. This set of constraints states that the total flow in all paths between a given origin–destination must equal the demand of that origin–destination. At each stage of the assignment, the network link's capacity changes with different percentages of the presence of AVs, which is calculated through Eq. (1)^45^.

#### SO

This problem differs from UE problem only in the objective function, and at the same time, its objective function has a special change, unlike the previous problem. But the limitations are still the same as the previous problem. If the flow in each of the network links is multiplied by its travel time and the values on all the links of the network are added, the resulting expression will be the total travel time spent in the network according to Eq. (2)^46^.

2 $$\widetilde{Z}\left(X\right)= \sum_{a}{x}_{a}\cdot {t}_{a}\left({x}_{a}\right),$$

where ${t}_{a}\left({x}_{a}\right)$ is the average travel time for a vehicle in link $a$. This quantity, i.e. the total time spent in the system, can be important from the point of view of the operators of the transportation network system. While it is assumed that each person chooses a route in such a way as to improve the travel time. For network operators, the total travel time spent in the network will be more important than the interests of individual users, and this quantity can be a measure to evaluate the improvement or deterioration of the network condition from the point of view of its suppliers. The minimization problem of Eq. (3) is called SO problem^47^:

3 $${\text{min}}\widetilde{Z}\left(X\right)= {\sum }_{a}{x}_{a}\cdot {t}_{a}\left({x}_{a}\right),$$

$${\sum }_{k}{f}_{k}^{rs}= {q}_{rs} \forall rs$$

$${x}_{a}= {\sum }_{rs}{\sum }_{k}{f}_{k}^{rs}{\delta }_{a.k}^{rs} \forall a$$

$${f}_{k}^{rs} \ge 0 \forall k. rs$$

The flow pattern resulting from this problem is called SO flow pattern. This flow pattern may not necessarily match UE flow pattern because the normal flow pattern is defined so that each individual minimizes his own travel time, not that all travelers cumulatively decide to minimize the network travel time. Obviously, the system operator would like to observe SO flow patterns in the network, but normally such a wish will not be realized because this flow pattern may not necessarily match the normal flow pattern resulting from individual users' decisions. In fact, it can be said that in the normal case, obtaining SO flow pattern will require that several network passengers increase their personal travel time from the minimum state in favor of the total travel time of the network. It is clear that this will not happen normally. For this reason, an issue called the imposition of tolls, or road taxes, can be raised in transportation networks. That is, the operator of the system and certain routes or links places taxes or tolls that move the flow in the network towards the desired pattern, which is SO flow pattern^48^.

#### Travel time function

Suppose, in the study of a highway network, there is a function for each link that expresses the relationship between safety and traffic volume. The Bureau of Public Roads (BPR) developed the link congestion function (delay-volume, or link performance, travel time, or cost), which is shown in Eq. (4)^43^:

4 $${t}_{a}\left({x}_{a}\right)= {t}_{a}^{f}\left(1+0.15{\left(\frac{{x}_{a}}{{c}_{a}}\right)}^{4}\right),$$

where ${t}_{a}^{f}$ is the free-flow travel time on link $a$, ${c}_{a}$ is the capacity of link $a$. The travel time function at low volumes usually has a very small slope, and its sensitivity to flow is small. Therefore, in such volumes, the travel time of network links is almost independent of the volume, so the normal flow pattern (UE) will almost match the optimal flow pattern. Therefore, at lower densities, the users and system operator benefit is almost compatible with each other, and with the increase in traffic volume and the growth of congestion, these two conflict with each other. Now, since in this research, both SO and UE assignments are done simultaneously, it can be used as a cost function to solve SO problem by deriving the time function. If the travel time functions in network links are given as Eq. (5), solving UE problem with these travel times will yield the same SO of the system.

5 $${\widetilde{t}}_{a}\left({x}_{a}\right)={t}_{a}\left({x}_{a}\right)+{x}_{a}\frac{d{t}_{a}({x}_{a})}{d{x}_{a}}.$$

Also, if SO problem is solved with travel time functions as Eq. (6), the resulting solution will be the same as the solution of UE problem^49^.

6 $${\widehat{t}}_{a}\left({x}_{a}\right)=\frac{1}{{x}_{a}}{\int }_{0}^{{x}_{a}}{t}_{a}\left(\omega \right)d\omega .$$

#### Frank-Wolfe algorithm

The Frank-Wolfe algorithm is a first-order iteration algorithm for constrained convex optimization. In each iteration, the Frank-Wolfe algorithm considers a linear approximation of the objective function and moves towards the minimization of this linear function. This method was originally proposed by Marguerite Frank and Philip Wolfe in 1956. Solving the Frank-Wolfe convex combination algorithm problem is shown step by step as follows^50–52^:

*Step 0*: Finding a feasible solution ${X}_{0}$

*Step 1*: Finding ${Y}^{n}$ by solving the problem in Eq. (7):

7 $$Min\overrightarrow{ \nabla Z\left({X}^{n }\right)\cdot } \overrightarrow{{Y}^{n}},$$

$$s\cdot t\cdot \sum_{i}{h}_{ij}{Y}_{i}^{n}\ge {b}_{j} \forall j\in {j}^{n}$$

where $\nabla Z\left({X}^{n}\right)$ denotes the gradient of $Z$ evaluated at ${X}^{n}$, ${X}^{n}$ is link flows at iteration $n$, ${Y}^{n}$ is an auxiliary flow pattern, i and j are indices of origin and destination locations, respectively, ${h}_{ij}$ is the coefficient matrix in link $i-j$, and ${b}_{j}$ is the right hand side vector.

*Step 2*: Finding step length ${a}_{n}$ by solving the problem in Eq. (8):

8 $$Min Z\left(\left(1-{a}_{n}\right){X}^{n}+{a}_{n}{Y}^{n} \right),$$

$$s\cdot t\cdot 0\le {a}_{n}\le 1$$

*Step 3*: Finding the new point (as indicated in Eq. (9)):

9 $${X}^{n+1}=\left(1-{a}_{n}\right){X}^{n}+{a}_{n}{Y}^{n}.$$

*Step 4*: If the termination rule is valid, end. Otherwise, 1 + n → n and go to step 1 and repeat the problem again.

Frank Wolfe's algorithm tries to choose $n$ more intelligently: in each iteration, $n$ is chosen so that along the connecting line, the link flow $X$ to the optimal solution ${X}^{*}$ is as close to equilibrium as possible^44^.

#### Method of successive averages (MSA)

In choosing the value of $n$, there may be two wrong situations. If $n$ is "too large", overcorrection occurs (and may oscillate continuously). If it is "too small", it will take too long to complete (if there is one). MSA tries to avoid both errors by starting with large values of $n$ and moving towards smaller values. If ${n}_{i}$ is the size of each step for the ith iteration (as Eq. 10):

10 $${n}_{i}=\frac{1}{k} k=2. 3.$$

According to the above explanations, it should be noted that this method differs from the Frank Wolfe algorithm only in step 2.

### Accessibility measurement

The final goal of this research is to measure the impact of the presence of AVs on the accessibility of the transportation network. Therefore, in this regard, in the continuation of the written codes after the end of each assignment, the amount of accessibility is measured and recorded by two relations of gravity and accessibility index.

#### Gravity

A road network is represented by a directed graph $g=(N.E)$, where $N$ is the set of nodes and $E$ represents the set of links. Each link $e$ in the set $E$ $(e\in E)$ has a travel time depending on the flow ${t}_{e}={t}_{{(y}_{e})}$, where ${(y}_{e})$ is the flow of the link, and $t(\cdot )$ is the travel time function of the link. If ${p}_{k}^{rs}$ is a path between the pair of origin and destination (O-D), the travel time of the path ${t}_{k}^{rs}$ is calculated as the result of all the travel times of the links along the path in the form of Eq. (11)^53^:

11 $${t}_{k}^{rs}= \sum_{e}{t}_{e}{\delta }_{k.e}^{rs} .$$

In which, ${\delta }_{k.e}^{rs}$ is the random index of the link path; If the path ${p}_{k}^{rs}$ passes through link $e$, ${\delta }_{k.e}^{rs}=1$, and otherwise, it is equal to zero. This means that ${P}_{rs}$ is the shortest route among all routes with the shortest travel time ${t}_{rs}$ between two points of origin and destination.

If $(\dots . {f}_{j}^{s}.\dots )$ is a set of urban opportunities or facilities (such as businesses, shops, etc.) at destination $s$, based on the concept of gravity-based accessibility, the accessibility ${A}_{rs}$ of people living in a place $r$ for their participation in activities from destination $s$ is formulated in the form of Eq. (12):

12 $${A}_{rs}= \sum_{\forall f{i}^{s}}{w}_{f{i}^{s}}{\text{exp}}\left(-\alpha {t}_{rs}\right)= exp\left(-\alpha {t}_{rs}\right)\sum_{\forall {f}_{i}^{s}}{w}_{{f}_{i}^{s}}.$$

In which, ${w}_{f{i}^{s}}$ is the attractiveness of ${f}_{i}^{s}$ facility and reflects the service quality of this facility (such as size, service price, etc.) in destination $s$. The negative exponential function ${\text{exp}}\left(-\alpha {t}_{rs}\right)$ is assumed as the continuity function, and $\alpha$ is also used as the calibration parameter. Various experimental studies have indicated that the negative exponential function is superior to other functions, such as the inverse power function, in estimating transport flows. The considered calibration parameter is 0.385^54,55^.

If ${w}_{s}= {\sum }_{\forall f{i}^{s}}{w}_{f{i}^{s}}$ is equivalent to the attractiveness of all facilities in the destination, and considering ${w}_{s}$ in Eq. (12), accessibility ${A}_{rs}$ is described in the form of Eq. (13):

13 $${A}_{rs}= {w}_{s}exp\left(-\alpha {t}_{rs}\right) .$$

The sum of ${A}_{rs}$ for all destinations results in the accessibility of region $r$, denoted by ${A}_{r}$ as Eq. (14):

14 $${A}_{r}=\sum_{\forall s}{A}_{rs}= \sum_{\forall s}{w}_{s}exp\left(-\alpha {t}_{rs}\right).$$

Accessibility ${A}_{r}$ refers to the ease (or ability) of people living in location $r$ to access their desired activities at a specific destination through the road network. It can be an effective measure to evaluate the level of social services provided to people in the area.

If ${O}_{r}$ represents the population of region r, the total system accessibility, denoted by $SA$, is as Eq. (15):

15 $$SA= \sum_{\forall r}{O}_{r}{A}_{r}.$$

This index represents the accessibility provided to the entire population in the gravity area^56^.

#### Accessibility index

The amount of accessibility defined in this section is based on the total cost, which includes the travel time and the imposed cost resulting from applying the selective demand management policy, which is obtained to bring the network into equilibrium^57^. The accessibility index (${IA}_{ij}$) for each area like $j$ is defined in the form of Eq. (16):

16 $${IA}_{ij}=\frac{\sum_{a}^{n}{\delta }_{aj}{x}_{a}/[{t}_{a}({x}_{a})+{\tau }_{a}]}{n} {\forall }_{ij {\prime}}a\in A{A}_{j {\prime}} ij\in rs$$

where $A{A}_{j}$ indicates all the links that end in area $j$, ${\tau }_{a}$ is the link-based cost on link $a$, and ${\delta }_{aj}=\left\{\genfrac{}{}{0pt}{}{1}{0}\right\}$. If the path to access area $j$ includes link $a$, ${\delta }_{aj}=1$, and otherwise, ${\delta }_{aj}=0$.

In Eq. (16), the accessibility index is practically defined for each area. From this point of view, the accessibility index for the whole network (${IA}_{T}$) is defined according to Eq. (17):

17 $${IA}_{T}=\sum_{J}^{n}{A}_{j},$$

where ${A}_{j}$ stands for the accessibility of the jth zone.

Based on Eq. (17), it is possible to define Eq. (18) to maximize the amount of accessibility in the network. This objective function is practically at the highest level of the model and maximizes the accessibility index for the entire network:

18 $${\forall }_{ij}, a\in A, i j \in rs MAX {Z}_{3}= \sum_{j}{A}_{j }.$$

### Applying the created data of the networks in the database to the codes

After writing the appropriate codes, the data prepared based on Hearn and Sioux Falls network is called from the relevant library in the system. The library is the database that is stored in the name of each transportation network in the system, and the necessary data is collected in it.

### Implementation of transport network assignment codes in Anaconda software

The coding platform in this research is Anaconda software. In this section, a brief explanation is presented about this software. Anaconda is a widely used data science platform that comes with many tools. To put it more simply, Anaconda is a package full of useful and widely used tools for programming languages, Python, etc., which are mostly used in data science. In fact, Anaconda includes the main Python language, 100 + Python packages (libraries), compilers or editors (such as Pycharm), and Jupiter and Conda, which manages Anaconda's own packages. The purpose of code execution is to implement these codes written in the software and get the appropriate output from these codes.

### Changing the constant demand distribution of the network between UE and SO

In order to achieve the optimum state in the assignment, the amount of constant demand is divided between two modes of AVs and non-AVs in different proportions. For this purpose, for AVs, the assignment is performed in SO method, and for non-AVs, this assignment is conducted in the form of UE. Assignment of this amount of demand continues until the optimum state of both types of assignment is realized.

### Capacity assignment scenarios

In this study, three scenarios for calculating different capacity modes in the network have been investigated.

#### First scenario

In this scenario, the network capacity is assumed to be constant, and the assignment is done without changing the assumed network capacity.

#### Second scenario

In this scenario, the capacity is also changed after changing the percentage of AVs, and then the assignment is conducted.

#### Third scenario

In this scenario, after changing the percentage of AVs in the entire network at each stage, the percentage assignment of these vehicles in all network links is calculated separately, and the capacity of each link is obtained, and then the assignment is repeated until convergence is achieved.

### Process of advancing the issue

**Figure 2:**
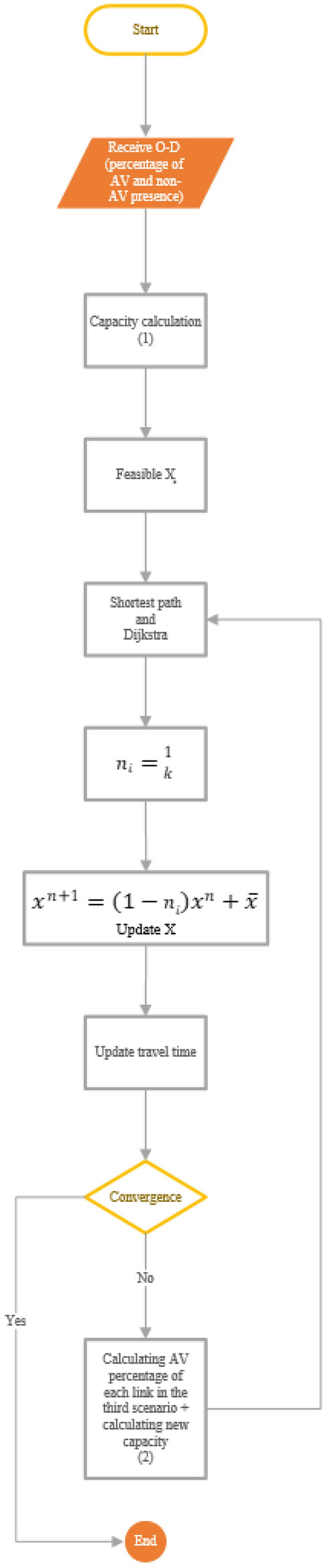
Flowchart of work progress.

Figure 2 shows the study’s progress from a mathematical point of view.

*Step 1*: Receiving the input, i.e. network specifications and origin–destination matrix, which determines the percentage of AVs and non-AVs in the network.

*Step 2*: Calculation of new network capacity (${C}_{m}$) using Eq. (19):

19 $${C}_{m}: \frac{v}{{\eta }^{2}v{T}_{aa}+\eta \left(1-\eta \right)v{T}_{ah}+\left(1-\eta \right)v{T}_{hx}+L},$$

where $v$ is the average speed, $L$ is the length of a vehicle, $\eta$ is the share of AVs in the total volume, ${T}_{aa}$, ${T}_{ah}$ and ${T}_{hx}$, respectively, are time gaps between AVs, AV and preceding vehicle, and other vehicles. As realistic values for the headways, ${T}_{aa}$ = 0.5 s, ${T}_{ah}$ = 0.9 s, and ${T}_{hx}$ = 1.15 s can be used^49^.

*Step 3*: In this step, the first stage of the assignment is done.

*Step 4*: Finding the shortest path and solving the Dijkstra problem.

*Step 5*: This step is obtained by dividing 1 by the iteration number. In fact, this step is the only difference between MSA and Frank Wolfe methods.

*Step 6*: This step is the third stage of the assignment, where $x$ is updated.

*Step 7*: In this step, the travel time is updated. The travel time of AVs and non-AVs is calculated separately through UE and SO relations.

*Step 8*: Convergence check: In this step, if convergence occurs, the final result has been achieved, and the solution to the problem ends. Otherwise, if the third scenario is followed, it is referred to the ninth step, and if the second scenario is used, it is referred to the fourth step.

*Step 9*: Calculating the new percentage of AVs and non-AVs in each link according to the allocation in Eq. (20) and calculating the new capacity, and returning to the fourth step. This cycle continues until convergence takes place. The first scenario will be obtained if steps two and nine are removed from the cycle.

20 $${\eta }_{i-j}^{AV}=\frac{{x}_{i-j}^{AV}}{{x}_{i-j}^{AV}+ {x}_{i-j}^{HV}},$$

where ${\eta }_{i-j}^{AV}$ is the share of AVs in the total volume in link $i-j$, ${x}_{i-j}^{AV}$ is the flow of AVs in link $i-j$, ${x}_{i-j}^{HV}$ is the flow of non-AVs in link $i-j$.

### Simultaneous assignment of AVs and regular vehicles

In order to assign AVs, different solutions can be considered. But as it is assumed in this study, AVs will determine their chosen route by the system. In this way, the chosen path of AVs must be in line with SO, while regular vehicles follow UE. Of course, this assumption will be implemented when the system operators consider the benefits for the users of AVs. These benefits, which are awarded in exchange for the mandatory route determination by the system operators for AVs, can be considered when purchasing a vehicle, annual tax, exclusive lanes, or road tolls for this type of vehicle users. In fact, considering these benefits, the fairness between the users of AVs and regular vehicles can be considered. Otherwise, the possibility of choosing AVs will be greatly reduced.

According to the mentioned assumption, a solution is provided for assignment with these features so that these two groups of vehicles can be assigned simultaneously with two separate travel time functions. It should be noted that travel time functions can be more than two states. However, in this study, two states of the travel time function in UE and SO states have been considered, which are actually the derivative of the travel time function in UE state.

The proposed solution is the same as a simple assignment, with the difference that some parts of the assignment algorithm are separated. In the code represented in Eq. (21), this solution is displayed:21where ${\widehat{x}}_{av}$ *and* ${\widehat{x}}_{pv}$ are the flow value of autonomous and passenger vehicles (${x}_{av}$ and ${x}_{pv}$), respectively, in the network resulting from the all-or-none assignment, *G* and* p* are Dijkstra origin and destination, respectively, ${d}_{rs}^{av}$ and ${d}_{rs}^{pv}$ are travel demands for autonomous and passenger vehicles, respectively, $SPTT$ is the shortest path travel time, ${SPTT}^{av}$ and ${SPTT}^{pv}$ are SPTT for autonomous and passenger vehicles, respectively, $TSTT$ is total system travel time, and ${TSTT}^{av}$ and ${TSTT}^{pv}$ are TSTT for autonomous and passenger vehicles, respectively.

Convergence value has no unit and no intuitive meaning. According to MSA method to calculate the convergence value, at the beginning of each iteration, the total system travel time can be calculated through Eq. (22):

22 $$TSTT={\sum }_{(i.j)\in A}{X}_{ij }{t}_{ij({x}_{ij})},$$

where ${t}_{ij({x}_{ij})}$ is the travel time for link $i-j$. Also, the shortest path travel time is calculated through Eq. (23):

23 $$SPTT={\sum }_{(i.j)\in A}{\widehat{X}}_{ij }{t}_{ij\left({x}_{ij}\right).}$$

It is worth mentioning:

24 $$SPTT<TSTT.$$

## Results

In this section, the software results and outputs are presented. In this regard, two examples with manual calculations were examined to measure accessibility in Hearn and Sioux Falls networks. It should be noted that these calculations were done for verification purposes. Also, the data of both investigated networks are expressed in the form of tables.

### Hearn network

**Figure 3:**
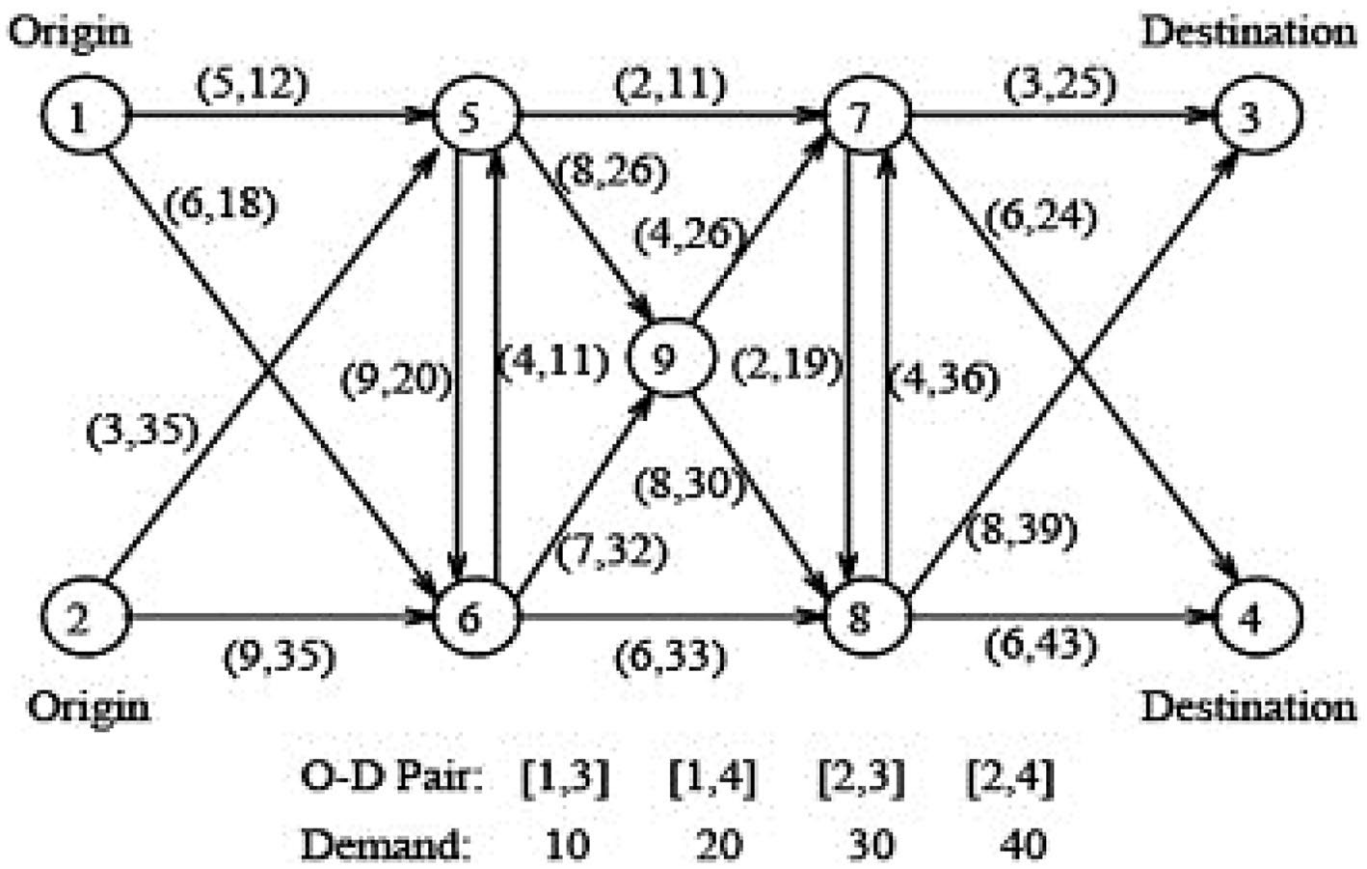
Hearn network.

In this section, the nine-node Hearn network was used to solve the example, which was applied in many types of research as a test sample with data similar to the large-scale traffic assignment problem. The topology of the considered network is shown in Fig. 3^58,59^.

As shown in Fig. 3, the network has nine nodes, eighteen links with the same functional structure, and four origin–destination pairs: [1, 1–3, 3, 4], and [2, 4]. As shown at the bottom of the figure, the demand from node 1 to node 3, node 1 to node 4, node 2 to node 3, and node 2 to node 4 is 10, 20, 30, and 40 units, respectively. The general data of Hearn network is presented in Table 1.

**Table 1:** Hearn network data.

Origin	Destination	Capacity	Length	Free flow travel time	Origin
1	5	12	5	5	1
1	6	18	6	6	1
2	5	35	3	3	2
2	6	35	9	9	2
5	6	20	9	9	5
5	7	11	2	2	5
5	9	26	8	8	5
6	5	11	4	4	6
6	8	33	6	6	6
6	9	32	7	7	6
7	3	25	3	3	7
7	4	24	6	6	7
7	8	19	2	2	7
8	3	39	8	8	8
8	4	43	6	6	8
8	7	36	4	4	8

#### Numerical example on Hearn network

In this section, the numerical solution of the accessibility index in two destinations 3 and 4 of the Hearn network are presented in the two cases of the absence of AVs and 100% presence of AVs according to the accessibility index relations (Eqs. 16 and 17). The calculation of accessibility in the absence and presence of AVs is presented in Supplementary Eqs. (25) and (26), respectively.

#### Results of the scenarios in Hearn network

**Figure 4:**
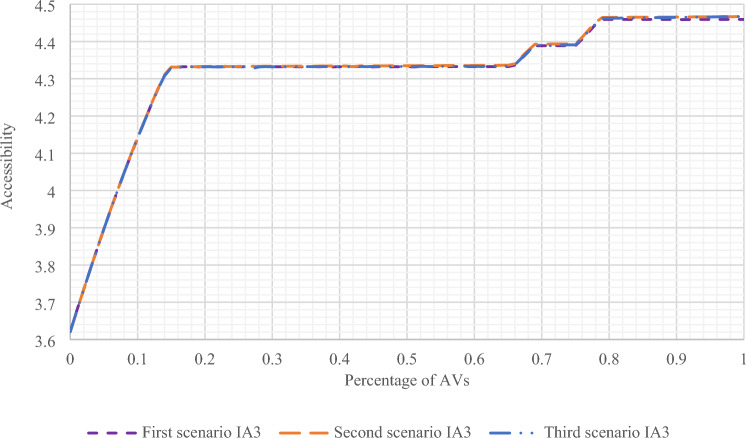
The amount of accessibility index in destination 3 in all three scenarios with a change in the presence percentage of AVs.

**Figure 5:**
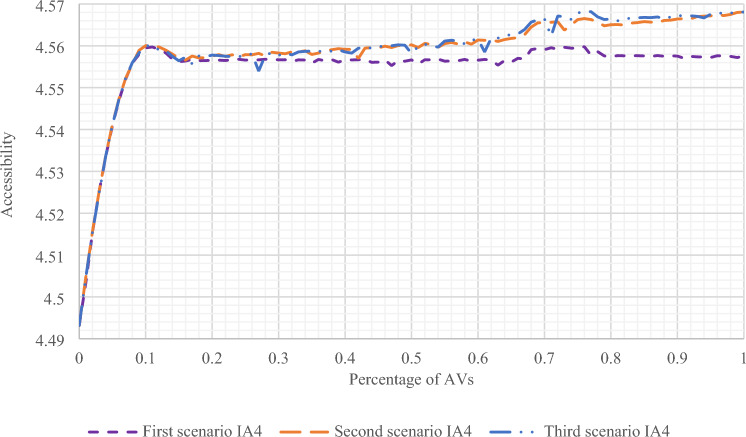
The amount of accessibility index in destination 4 in all three scenarios with a change in the presence percentage of AVs.

**Figure 6:**
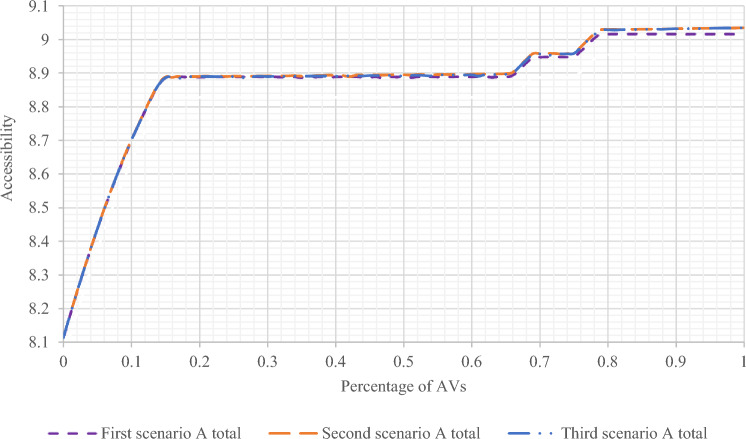
The amount of accessibility index of the entire Hearn network in all three scenarios with a change in the presence percentage of AVs.

In this section, the results of all three scenarios of Hearn network were presented and compared. As can be seen in Figs. 4, 5 and 6, in all three scenarios, the accessibility index increases, but in the second and third scenarios, this increase in the accessibility index is more significant due to the change in the capacity of the entire network.

In all scenarios, according to the fact that there are two destinations 3 and 4 of the Hearn network, it can be seen in Figs. 4 and 5 that the network accessibility index increases with the increase in the presence of AVs in each destination separately. As a result, this accessibility has increased in the total network. As shown in Fig. 6, this increase is steep in the first 20 percent of the presence of AVs and then gradually increases. Also, in the first scenario, the capacity is constant and does not differ from the constant capacity of the network. That is, with the increase in the presence of AVs, there has been no change in the network capacity. It is worth noting that in this network, due to the lack of data, including the population, as well as the small size of the network, only the accessibility index has been investigated, and the gravity-based accessibility has not been examined. On the other hand, in the second scenario, the network capacity changes with different percentages of the presence of AVs. Moreover, in the third scenario, after an assignment stage, the percentage of presence of AVs in all links is calculated, and then the capacity is changed again.

### Sioux Falls network

**Figure 7:**
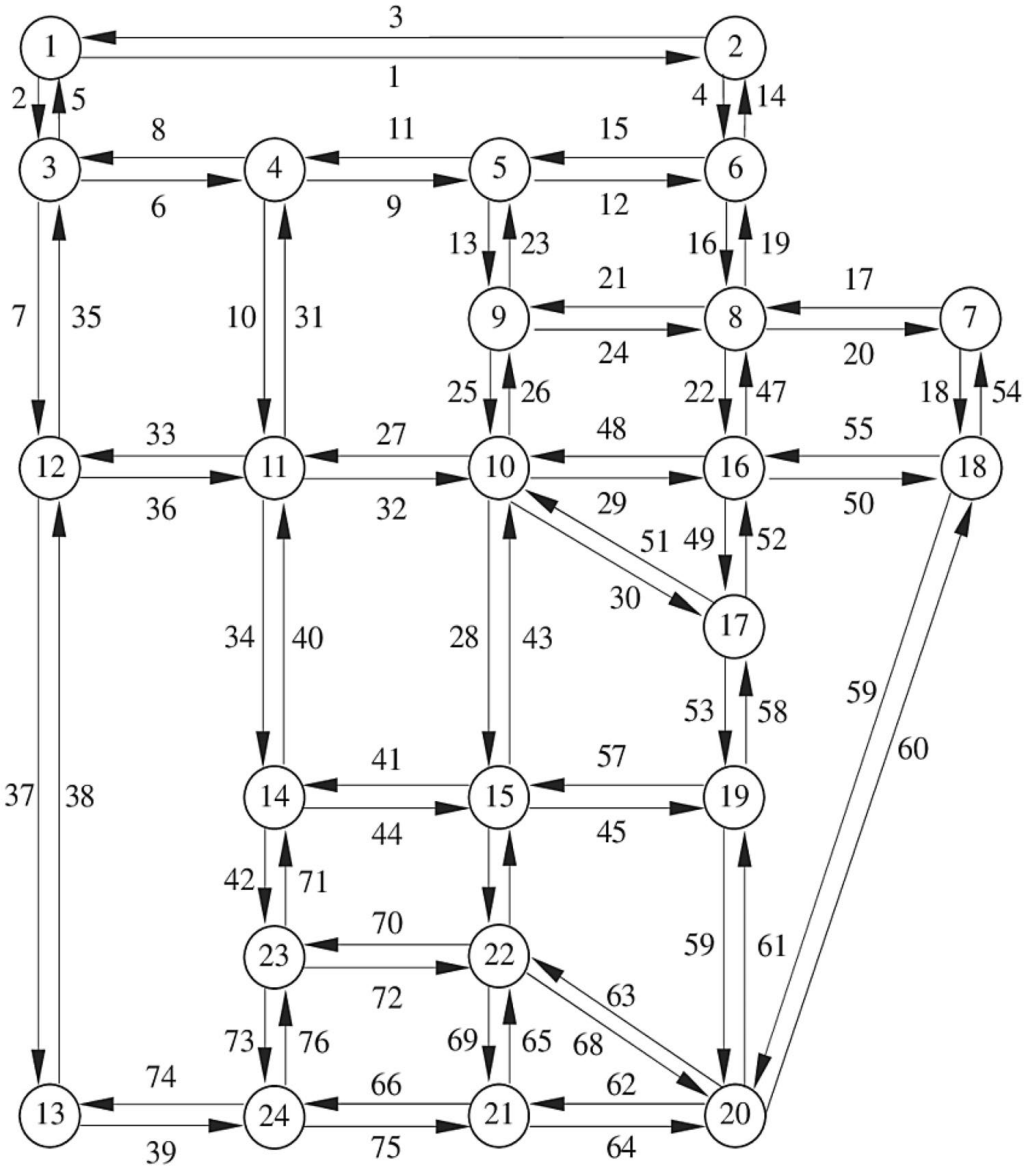
Sioux Falls transportation network.

Sioux Falls transportation network is a network located in the state of South Dakota, the United States of America, which includes 24 nodes and 76 links, as indicated in Fig. 7. For the simulation of potential impacts of autonomous vehicles (AVs), we selected the Sioux Falls transportation network. This decision was motivated by several factors. Primarily, we aimed to apply our model to real-world examples to comprehensively understand the implications of AV adoption in actual urban environments. Sioux Falls, a small mid-western US city, offers a unique opportunity to explore AV impacts in settings differing from the typical large metropolitan areas often studied in the literature. Additionally, examining AV effects in smaller cities and rural areas, where transportation options may be limited, is crucial. By selecting Sioux Falls, we aimed to elucidate the potential benefits and challenges of AV deployment in such regions, where transit and alternative transportation modes might be less available.

Moreover, the Sioux Falls transportation network provides several advantages for research purposes. Its small-scale and relatively simple structure, with only 24 nodes and 76 links, facilitate analysis and modeling compared to larger, more complex networks. Furthermore, the extensive utilization of the Sioux Falls network in transportation literature offers abundant data and research for comparison and benchmarking purposes. The network has served various research objectives, including evacuation planning, traffic congestion management, and network design, making it a versatile test case for our study.

In summary, the Sioux Falls transportation network serves as a valuable resource for transportation researchers, enabling the testing and refinement of new models and algorithms in a controlled and well-understood setting.

Tables 2 and 3 present the details of the Sioux Falls network. Due to the lack of data and characteristics of the population in Sioux Falls network, the data has been used from the study of Yan et al., who calculated values for the population and other characteristics based on the demand^60^. Also, the speed values and the number of lanes are assumed in this research. The general characteristics of Sioux Falls network and the amount of origin–destination demand in this network are presented in Tables 2 and 3, respectively.

**Table 2:** General characteristics of Sioux Falls network.

Origin	Destination	Number of lanes	Attractiveness	Population	Speed	Free flow travel time	Length	Capacity
1	2	0	0.195	14,667	25	6	6	25,900.0000
1	3	3	0.195	14,667	50	4	4	23,403.47319
2	1	3	0.089	6667	50	6	6	25,900.20064
2	6	2	0.089	6667	25	5	5	4958.180928
3	1	2	0.062	4667	25	4	4	23,403.47319
3	4	2	0.062	4667	25	4	4	17,110.52372
3	12	2	0.062	4667	25	4	4	23,403.47319
4	3	1	0.259	19,333	25	4	4	17,110.52372
4	5	1	0.259	19,333	25	2	2	17,782.7941
4	11	2	0.259	19,333	25	6	6	4908.82673
5	4	2	0.135	10,167	25	2	2	17,782.7941
5	6	2	0.135	10,167	25	4	4	4947.995469
5	9	2	0.135	10,167	25	5	5	10,000
6	2	3	0.169	12,667	50	5	5	4958.180928
6	5	3	0.169	12,667	50	4	4	4947.995469
6	8	1	0.169	12,667	25	2	2	4898.587646
7	8	1	0.268	20,167	25	3	3	7841.81131
7	18	3	0.268	20,167	50	2	2	23,403.47319
8	6	3	0.370	27,833	50	2	2	4898.587646
8	7	2	0.370	27,833	25	3	3	7841.81131
8	9	2	0.370	27,833	25	10	10	5050.193156
8	16	2	0.370	27,833	25	5	5	5045.822583
9	5	2	0.361	20,250	25	5	5	10,000
9	8	2	0.361	20,250	25	10	10	5050.193156
9	10	1	0.361	20,250	25	3	3	13,915.78842
10	9	1	1	45,200	25	3	3	13,915.78842
10	11	2	1	45,200	25	5	5	10,000
10	15	2	1	45,200	25	6	6	13,512.00155
10	16	2	1	45,200	25	4	4	4854.917717
10	17	3	1	45,200	50	8	8	4993.510694
11	4	3	0.497	27,875	50	6	6	4908.82673
11	10	1	0.497	27,875	25	5	5	10,000
11	12	1	0.497	27,875	25	6	6	4908.82673
11	14	3	0.497	27,875	50	4	4	4876.508287
12	3	2	0.310	23,167	25	4	4	23,403.47319
12	11	1	0.310	23,167	25	6	6	4908.82673
12	13	2	0.310	23,167	25	3	3	25,900.20064
13	12	3	0.322	24,333	50	3	3	25,900.20064
13	24	3	0.322	24,333	50	4	4	5091.256152
14	11	2	0.313	23,500	25	4	4	4876.508287
14	15	2	0.313	23,500	25	5	5	5127.526119
14	23	2	0.313	23,500	25	4	4	4924.790605
15	10	1	0.472	26,750	25	6	6	13,512.00155
15	14	2	0.472	26,750	25	5	5	5127.526119
15	19	2	0.472	26,750	25	3	3	14,564.75315
15	22	2	0.472	26,750	25	3	3	9599.180565
16	8	2	0.579	32,625	25	5	5	5045.822583
16	10	2	0.579	32,625	25	4	4	4854.917717
16	17	3	0.579	32,625	50	2	2	5229.910063
16	18	1	0.579	32,625	25	3	3	19,679.89671
17	10	1	0.519	29,250	25	8	8	4993.510694
17	16	3	0.519	29,250	50	2	2	5229.910063
17	19	3	0.519	29,250	50	2	2	4823.950831
18	7	2	0.104	8000	25	2	2	23,403.47319
18	16	1	0.104	8000	25	3	3	19,679.89671
18	20	2	0.104	8000	25	4	4	23,403.47319
19	15	3	0.284	21,333	50	3	3	14,564.75315
19	17	1	0.284	21,333	25	2	2	4823.950831
19	20	2	0.284	21,333	25	4	4	5002.607563
20	18	2	0.408	30,833	25	4	4	23,403.47319
20	19	2	0.408	30,833	25	4	4	5002.607563
20	21	2	0.408	30,833	25	6	6	5059.91234
20	22	2	0.408	30,833	25	5	5	5075.697193
21	20	2	0.244	18,333	25	6	6	5059.91234
21	22	2	0.244	18,333	25	2	2	5229.910063
21	24	2	0.244	18,333	25	3	3	4885.357564
22	15	3	0.541	12,833	50	3	3	9599.180565
22	20	1	0.541	12,833	25	5	5	5075.697193
22	21	1	0.541	12,833	25	2	2	5229.910063
22	23	3	0.541	12,833	50	4	4	5000
23	14	3	0.322	24,167	50	4	4	4924.790605
23	22	2	0.322	24,167	25	4	4	5000
23	24	2	0.322	24,167	25	2	2	5078.508436
24	13	2	0.173	40,667	25	4	4	5091.256152
24	21	2	0.173	40,667	25	3	3	4885.357564
24	23	1	0.173	40,667	25	2	2	5078.508436

**Table 3:** The amount of origin–destination demand in Sioux Falls network.

Origin	Destination	Demand	Origin	Destination	Demand	Origin	Destination	Demand
1	2	100.0	2	10	600.0	3	18	0.0
1	3	100.0	2	11	200.0	3	19	0.0
1	4	500.0	2	12	100.0	3	20	0.0
1	5	200.0	2	13	300.0	3	21	0.0
1	6	300.0	2	14	100.0	3	22	100.0
1	7	500.0	2	15	100.0	3	23	100.0
1	8	800.0	2	16	400.0	3	24	0.0
1	9	500.0	2	17	200.0	4	1	500.0
1	10	1300.0	2	18	0.0	4	2	200.0
1	11	500.0	2	19	100.0	4	3	200.0
1	12	200.0	2	20	100.0	4	5	500.0
1	13	500.0	2	21	0.0	4	6	400.0
1	14	300.0	2	22	100.0	4	7	400.0
1	15	500.0	2	23	0.0	4	8	700.0
1	16	500.0	2	24	0.0	4	9	700.0
1	17	400.0	3	1	100.0	4	10	1200.0
1	18	100.0	3	2	100.0	4	11	1400.0
1	19	300.0	3	4	200.0	4	12	600.0
1	20	300.0	3	5	100.0	4	13	600.0
1	21	100.0	3	6	300.0	4	14	500.0
1	22	400.0	3	7	100.0	4	15	500.0
1	23	300.0	3	8	200.0	4	16	800.0
1	24	100.0	3	9	100.0	4	17	500.0
2	1	100.0	3	10	300.0	4	18	100.0
2	3	100.0	3	11	300.0	4	19	200.0
2	4	200.0	3	12	200.0	4	20	300.0
2	5	100.0	3	13	100.0	4	21	200.0
2	6	400.0	3	14	100.0	4	22	400.0
2	7	200.0	3	15	100.0	4	23	500.0
2	8	400.0	3	16	200.0	4	24	200.0
2	9	200.0	3	17	100.0	5	1	200.0
5	2	100.0	6	11	400.0	7	19	400.0
5	3	100.0	6	12	200.0	7	20	500.0
5	4	500.0	6	13	200.0	7	21	200.0
5	6	200.0	6	14	100.0	7	22	500.0
5	7	200.0	6	15	200.0	7	23	200.0
5	8	500.0	6	16	900.0	7	24	100.0
5	9	800.0	6	17	500.0	8	1	800.0
5	10	1000.0	6	18	100.0	8	2	400.0
5	11	500.0	6	19	200.0	8	3	200.0
5	12	200.0	6	20	300.0	8	4	700.0
5	13	200.0	6	21	100.0	8	5	500.0
5	14	100.0	6	22	200.0	8	6	800.0
5	15	200.0	6	23	100.0	8	7	1000.0
5	16	500.0	6	24	100.0	8	9	800.0
5	17	200.0	7	1	500.0	8	10	1600.0
5	18	0.0	7	2	200.0	8	11	800.0
5	19	100.0	7	3	100.0	8	12	600.0
5	20	100.0	7	4	400.0	8	13	600.0
5	21	100.0	7	5	200.0	8	14	400.0
5	22	200.0	7	6	400.0	8	15	600.0
5	23	100.0	7	8	1000.0	8	16	2200.0
5	24	0.0	7	9	600.0	8	17	1400.0
6	1	300.0	7	10	1900.0	8	18	300.0
6	2	400.0	7	11	500.0	8	19	700.0
6	3	300.0	7	12	700.0	8	20	900.0
6	4	400.0	7	13	400.0	8	21	400.0
6	5	200.0	7	14	200.0	8	22	500.0
6	7	400.0	7	15	500.0	8	23	300.0
6	8	800.0	7	16	1400.0	8	24	200.0
6	9	400.0	7	17	1000.0	9	1	500.0
6	10	800.0	7	18	200.0	9	2	200.0
9	3	100.0	10	12	2000.0	11	20	600.0
9	4	700.0	10	13	1900.0	11	21	400.0
9	5	800.0	10	14	2100.0	11	22	1100.0
9	6	400.0	10	15	4000.0	11	23	1300.0
9	7	600.0	10	16	4400.0	11	24	600.0
9	8	800.0	10	17	3900.0	12	1	200.0
9	10	2800.0	10	18	700.0	12	2	100.0
9	11	1400.0	10	19	1800.0	12	3	200.0
9	12	600.0	10	20	2500.0	12	4	600.0
9	13	600.0	10	21	1200.0	12	5	200.0
9	14	600.0	10	22	2600.0	12	6	200.0
9	15	900.0	10	23	1800.0	12	7	700.0
9	16	1400.0	10	24	800.0	12	8	600.0
9	17	900.0	11	1	500.0	12	9	600.0
9	18	200.0	11	2	200.0	12	10	2000.0
9	19	400.0	11	3	300.0	12	11	1400.0
9	20	600.0	11	4	1500.0	12	13	1300.0
9	21	300.0	11	5	500.0	12	14	700.0
9	22	700.0	11	6	400.0	12	15	700.0
9	23	500.0	11	7	500.0	12	16	700.0
9	24	200.0	11	8	800.0	12	17	600.0
10	1	1300.0	11	9	1400.0	12	18	200.0
10	2	600.0	11	10	3900.0	12	19	300.0
10	3	300.0	11	12	1400.0	12	20	400.0
10	4	1200.0	11	13	1000.0	12	21	300.0
10	5	1000.0	11	14	1600.0	12	22	700.0
10	6	800.0	11	15	1400.0	12	23	700.0
10	7	1900.0	11	16	1400.0	12	24	500.0
10	8	1600.0	11	17	1000.0	13	1	500.0
10	9	2800.0	11	18	100.0	13	2	300.0
10	11	4000.0	11	19	400.0	13	3	100.0
13	4	600.0	14	12	700.0	15	21	800.0
13	5	200.0	14	13	600.0	15	22	2600.0
13	6	200.0	14	15	1300.0	15	23	1000.0
13	7	400.0	14	16	700.0	15	24	400.0
13	8	600.0	14	17	700.0	16	1	500.0
13	9	600.0	14	18	100.0	16	2	400.0
13	10	1900.0	14	19	300.0	16	3	200.0
13	11	1000.0	14	20	500.0	16	4	800.0
13	12	1300.0	14	21	400.0	16	5	500.0
13	15	700.0	14	23	1100.0	16	7	1400.0
13	16	600.0	14	24	400.0	16	8	2200.0
13	17	500.0	15	1	500.0	16	9	1400.0
13	18	100.0	15	2	100.0	16	10	4400.0
13	19	300.0	15	3	100.0	16	11	1400.0
13	20	600.0	15	4	500.0	16	12	700.0
13	21	600.0	15	5	200.0	16	13	600.0
13	22	1300.0	15	6	200.0	16	14	700.0
13	23	800.0	15	7	500.0	16	15	1200.0
13	24	800.0	15	8	600.0	16	17	2800.0
14	1	300.0	15	9	1000.0	16	18	500.0
14	2	100.0	15	10	4000.0	16	19	1300.0
14	3	100.0	15	11	1400.0	16	20	1600.0
14	4	500.0	15	12	700.0	16	21	600.0
14	5	100.0	15	13	700.0	16	22	1200.0
14	6	100.0	15	14	1300.0	16	23	500.0
14	7	200.0	15	16	1200.0	16	24	300.0
14	8	400.0	15	17	1500.0	17	1	400.0
14	9	600.0	15	18	200.0	17	2	200.0
14	10	2100.0	15	19	800.0	17	3	100.0
14	11	1600.0	15	20	1100.0	17	4	500.0
17	5	200.0	18	13	100.0	19	22	1200.0
17	6	500.0	18	14	100.0	19	23	300.0
17	7	1000.0	18	15	200.0	19	24	100.0
17	8	1400.0	18	16	500.0	20	1	300.0
17	9	900.0	18	17	600.0	20	2	100.0
17	10	3900.0	18	19	300.0	20	3	0.0
17	11	1000.0	18	20	400.0	20	4	300.0
17	12	600.0	18	21	100.0	20	5	100.0
17	13	500.0	18	22	300.0	20	6	300.0
17	14	700.0	18	23	100.0	20	7	500.0
17	15	1500.0	18	24	0.0	20	8	900.0
17	16	2800.0	19	1	300.0	20	9	600.0
17	18	600.0	19	2	100.0	20	10	2500.0
17	19	1700.0	19	3	0.0	20	11	600.0
17	20	1700.0	19	4	200.0	20	12	500.0
17	21	600.0	19	5	100.0	20	13	600.0
17	22	1700.0	19	6	200.0	20	14	500.0
17	23	600.0	19	7	400.0	20	15	1100.0
17	24	300.0	19	8	700.0	20	16	1600.0
18	1	100.0	19	9	400.0	20	17	1700.0
18	2	0.0	19	10	1800.0	20	18	400.0
18	3	0.0	19	11	400.0	20	19	1200.0
18	4	100.0	19	12	300.0	20	21	1200.0
18	5	0.0	19	13	300.0	20	22	2400.0
18	6	100.0	19	14	300.0	20	23	700.0
18	7	200.0	19	15	800.0	20	24	400.0
18	8	300.0	19	16	1300.0	21	1	100.0
18	9	200.0	19	17	1700.0	21	2	0.0
18	10	700.0	19	18	300.0	21	3	0.0
18	11	200.0	19	20	1200.0	21	4	200.0
18	12	200.0	19	21	400.0	21	5	100.0
21	6	100.0	22	14	1200.0	23	22	2100.0
21	7	200.0	22	15	2600.0	23	24	700.0
21	8	400.0	22	16	1200.0	24	1	100.0
21	9	300.0	22	17	1700.0	24	2	0.0
21	10	1200.0	22	18	300.0	24	3	0.0
21	11	400.0	22	19	1200.0	24	4	200.0
21	12	300.0	22	20	2400.0	24	5	0.0
21	13	600.0	22	21	1800.0	24	6	100.0
21	14	400.0	22	23	2100.0	24	7	100.0
21	15	800.0	22	24	1100.0	24	8	200.0
21	16	600.0	23	1	300.0	24	9	200.0
21	17	600.0	23	2	0.0	24	10	800.0
21	18	100.0	23	3	100.0	24	11	600.0
21	19	400.0	23	4	500.0	24	12	500.0
21	20	1200.0	23	5	100.0	24	13	700.0
21	22	1800.0	23	6	100.0	24	14	400.0
21	23	700.0	23	7	200.0	24	15	400.0
21	24	500.0	23	8	300.0	24	16	300.0
22	1	400.0	23	9	500.0	24	17	300.0
22	2	100.0	23	10	1800.0	24	18	0.0
22	3	100.0	23	11	1300.0	24	19	100.0
22	4	400.0	23	12	700.0	24	20	400.0
22	5	200.0	23	13	800.0	24	21	500.0
22	6	200.0	23	14	1100.0	24	22	1100.0
22	7	500.0	23	15	1000.0	24	23	700.0
22	8	500.0	23	16	500.0			
22	9	700.0	23	17	600.0			
22	10	2600.0	23	18	100.0			
22	11	1100.0	23	19	300.0			
22	12	700.0	23	20	700.0			
22	13	1300.0	23	21	700.0			

#### Numerical example on Sioux Falls network

The following example has been solved using the data obtained in the absence of AVs in the first scenario in Sioux Falls network. In this regard, the gravity-based accessibility amount of node 1 to all 24 nodes of this network according to Eqs. (13) and (14) is calculated as Supplementary Eq. (27):

#### Results of the scenarios in Sioux Falls network

**Figure 8:**
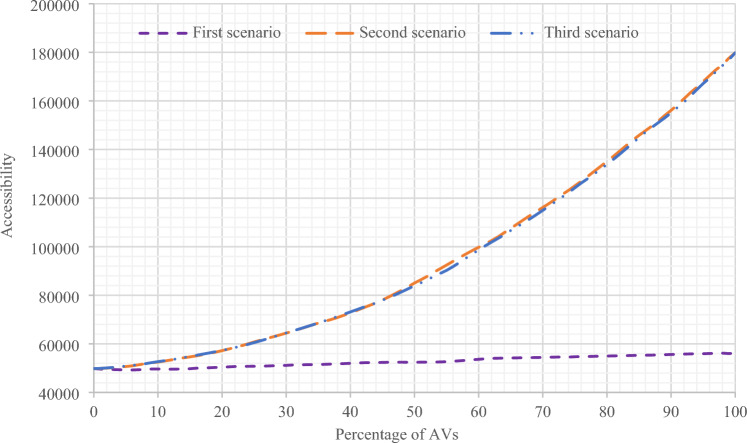
Comparing the results of the accessibility amount of all three scenarios with changes in the presence percentage of AVs using the gravity accessibility relation in Sioux Falls network.

**Figure 9:**
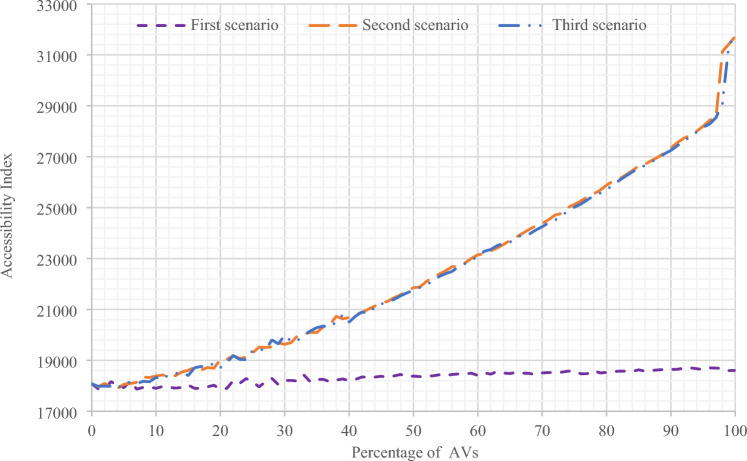
Comparing the results of the accessibility index of all three scenarios with changes in the presence percentage of AVs in Sioux Falls network.

In this section, the results of all three scenarios have been presented and compared in Sioux Falls network. In Figs. 8 and 9, the results of accessibility changes have been compared according to the changes in the percentage of presence of AVs in the network, based on three separate scenarios and in two states of gravity accessibility and accessibility index calculations. As can be seen, in the second and third scenarios, more significant increases in accessibility are evident compared to the first scenario.

As shown in Fig. 8 in the first scenario, the accessibility amount has increased with the increase in the presence of AVs after a slight recession in the range below 20% of the presence of AVs with a relatively constant slope. Also, in Fig. 9, for the first scenario, the same results can be seen by calculating the accessibility index. However, the difference is that there is no noticeable recession in the range below 20%. Moreover, in the second and third scenarios, the results indicating the increase in accessibility are visible in both figures.

**Figure 10:**
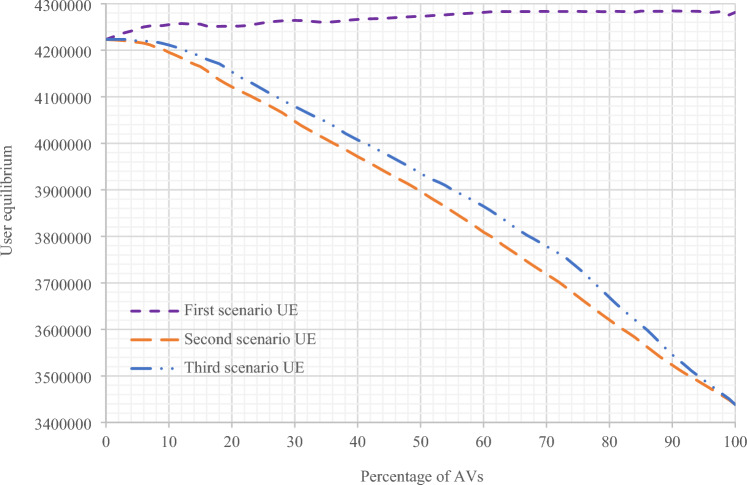
Comparing the results of UE objective functions in the assignment of all three scenarios with changes in the presence percentage of AVs in Sioux Falls network.

**Figure 11:**
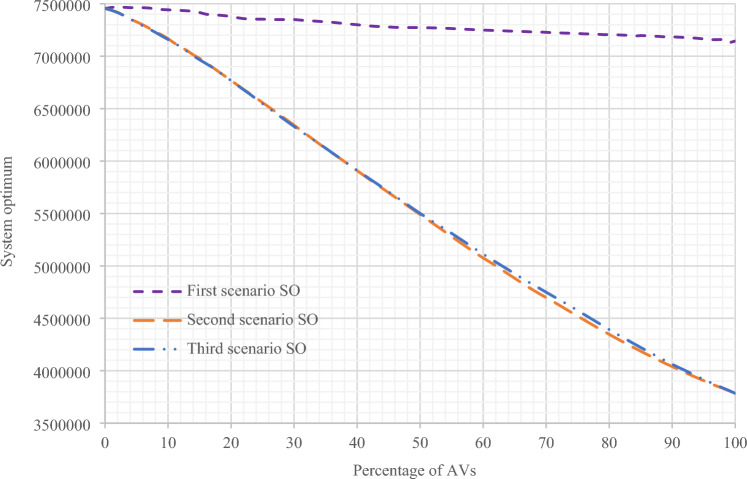
Comparing the results of SO objective functions in terms of vehicles per minute in all three scenarios with changes in the presence percentage of AVs in Sioux Falls network.

In Fig. 10, the comparison of the results of changes in the user equilibrium relation based on the changes in the percentage of presence of AVs in Sioux Falls network in the three scenarios proposed has been performed in the current research. Based on these results, it is evident that the user equilibrium increases in the first scenario, and in the next two scenarios, it decreases. This reduction in the user equilibrium is due to the increase in network capacity and its improvement. Also, in Fig. 11, the results of changes in the system optimum relation are compared based on the changes in the percentage of the presence of AVs in Sioux Falls network in the three proposed scenarios. Based on these results, in all three scenarios, the decrease in the results of system optimum function is evident because, with the presence of AVs, management by the system operators in the network increases, and the network system optimum tends to decrease. In the second and third scenarios, this reduction is more significant.

Also, as can be seen in Figs. 10 and 11 for the first scenario, as expected, the user equilibrium is facing an increase, and the system optimum graph has moved towards improvement (decrease). Because with the presence of a higher percentage of AVs, there is more management in the network and, accordingly, more vehicle control and route determination. On the other hand, in the second and third scenarios, due to the increase in network capacity at every stage of the assignment, with the increase in the percentage of the presence of AVs, there is a significant reduction in the results of the user equilibrium relation, in addition to the system optimum.

## Conclusion

In this study, an attempt has been made to analyze and investigate the impact of the presence of AVs on the accessibility of transportation networks by modeling in Hearn and Sioux Falls transportation networks as test networks. At first, a constant demand amount was assigned with different percentages of the presence of AVs in the network at the same time as non-AVs in the network. This assignment is coded with Python programming language. After examining the software inputs, it was observed that the results of the two equations of user equilibrium and system optimum had converged at each stage of the assignment, and then the assignment was finished, and from the obtained data, travel time was used to calculate accessibility based on a certain percentage of the presence of AVs. The results of this study showed that:

- With both states of accessibility calculation and in all scenarios, the accessibility trend is upward with the increase in the number of AVs.
- In both Hearn and Sioux Falls networks, the increase in accessibility in the second and third scenarios is higher than in the first scenario. This increase is due to the increase in the number of AVs and, as a result, the increase in capacity in the mentioned scenarios (second and third).
- As can be seen in the results of the first scenario of Sioux Falls network, with the presence of AVs in the network and the consequent increase in the management of the system operators in the network (determining the mandatory route for AVs), improvement in the system optimum function and increasing the user equilibrium is inevitable and evident in the results.
- Unlike the first scenario, in the second and third scenarios of Sioux Falls network, along with decreasing the system optimum objective function, the reduction of user equilibrium objective function is also observed. This decrease occurs due to the increase in capacity, which is because of the increase in the number of AVs in the network. Both reductions in the mentioned results have improved traffic flow assignment in the entire transportation network.

Moreover, it is suggested that future researchers in this field should also consider the following.

- Considering that, in reality, the total cost of travel changes with the collection of tolls in the network, it is necessary to examine the variability of demand in future studies.
- In the current research, the gravity and access index models were used to examine accessibility, so it is suggested to use the space–time method, which is another method of evaluating accessibility.
- Since various other data can be analyzed in different transportation networks, it is suggested that future research can be conducted based on those networks.
- The current research is based on the hybrid assignment algorithm. Since other hybrid assignment algorithms can also give different results, it is suggested to use the effects of other algorithms on accessibility and compare the results with the present research.

### Supplementary Information

Supplementary Information.

## Data Availability

Data are available from the corresponding author upon request.
