# Supplementary material for: Modeling the effect of autonomous vehicles (AVs) on the accessibility of the transportation network
Source: Sci Rep. 2024 Apr 23;14:9292. doi: 10.1038/s41598-024-60069-8 (PMC11039750; doi:10.1038/s41598-024-60069-8)
Supplement: Supplementary file 1 — Supplementary Information. [file 41598_2024_60069_MOESM1_ESM.docx]

**Supplementary Material**

Appendix

$A_{3}=\frac{\frac{X_{73}}{t_{73}}+\frac{X_{83}}{t_{83}}}{2}= \frac{\frac{38.15}{5.45}+\frac{1.84}{8}}{2}=3.6208$ (25)

$$A_{4}=\frac{\frac{X_{74}}{t_{74}}+\frac{X_{84}}{t_{84}}}{2}= \frac{\frac{17.37}{6.25}+\frac{42.62}{6.86}}{2}=4.493$$

$${IA}_{T}=3.628+4.493=8.1139$$

$A_{3}=\frac{\frac{X_{73}}{t_{73}}+\frac{X_{83}}{t_{83}}}{2}= \frac{\frac{29.6}{3.88}+\frac{10.4}{8}}{2}=4.464$ (26)

$$A_{4}=\frac{\frac{X_{74}}{t_{74}}+\frac{X_{84}}{t_{84}}}{2}= \frac{\frac{20.75}{6.5}+\frac{39.23}{6..62}}{2}=4.559$$

${IA}_{T}=4.464+4.559=9.023$

$A_{1.2}= 0.089\exp\left( -0.385\times6.0008 \right)=0.0088$ (27)

$$A_{1.3}= 0.062\exp\left( -0.385\times4.0086 \right)=0.013$$

$$A_{1.4}= 0.259\exp\left( -0.385\times8.2756 \right)=0.011$$

$$A_{1.5}= 0.135\exp\left( -0.385\times10.5895 \right)=0.0023$$

$$A_{1.6}= 0.169\exp\left( -0.385\times12.5688 \right)=0.0013$$

$$A_{1.7}= 0.268\exp\left( -0.385\times32.692 \right)=0.000000916$$

$$A_{1.8}= 0.37\exp\left( -0.385\times27.2056 \right)=0.0000105$$

$$A_{1.9}= 0.361\exp\left( -0.385\times20.2141 \right)=0.00015$$

$$A_{1.10}= 1\exp\left( -0.385\times25.888 \right)=0.0000469$$

$$A_{1.11}= 0.497\exp\left( -0.385\times15.411 \right)=0.0013$$

$$A_{1.12}= 0.31\exp\left( -0.385\times8.0286 \right)=0.0142$$

$$A_{1.13}= 0.322\exp\left( -0.385\times11.0514 \right)=0.0046$$

$$A_{1.14}= 0.313\exp\left( -0.385\times29.0663 \right)=0.00000432$$

$$A_{1.15}= 0.472\exp\left( -0.385\times39.5857 \right)=0.000000114$$

$$A_{1.16}= 0.579\exp\left( -0.385\times37.9049 \right)=0.000000266$$

$$A_{1.17}= 0.519\exp\left( -0.385\times42.1214 \right)=0.0000000470$$

$$A_{1.18}= 0.104\exp\left( -0.385\times34.7538 \right)=0.000000161$$

$$A_{1.19}= 0.284\exp\left( -0.385\times43.9062 \right)=0.0000000129$$

$$A_{1.20}= 0.408\exp\left( -0.385\times39.0122 \right)=0.000000122$$

$$A_{1.21}= 0.244\exp\left( -0.385\times40.3434 \right)=0.0000000438$$

$$A_{1.22}= 0.541\exp\left( -0.385\times44.5449 \right)=0.0000000193$$

$$A_{1.23}= 0.322\exp\left( -0.385\times32.3781 \right)=0.00000124$$

$$A_{1.24}= 0.173\exp\left( -0.385\times28.6558 \right)=0.0000028$$
